# Review of advances in CAR-T therapies in malignant hematology

**DOI:** 10.3389/fonc.2026.1768614

**Published:** 2026-03-17

**Authors:** Neda Amirmokhtari, Forat Lutfi

**Affiliations:** 1 Hematologic malignancies and cellular therapy, The University of Kansas Hospital - Westwood Medical Pavilion, Westwood, KS, United States; 2 Hematologic malignancies and cellular therapy, The University of Kansas Cancer Center Kansas City, Kansas, KS, United States

**Keywords:** acute lymphoblastic B-cell leukemia, adverse events, AES, anti-CD19 CAR-T therapy, auto-CARTs, lymphoma - diagnosis

## Abstract

There have been numerous advancements in immune cell effector therapies in the malignant hematology space. This review article explores the pivotal studies that led to the approval of chimeric antigen receptor T-cell (CAR-T) therapies for hematologic malignancies and highlights available associated long-term and real-world data. It also examines the mechanisms of resistance and potential novel therapies to overcome these mechanisms.

## 1 Introduction

Chimeric antigen receptor T-cell (CAR-T) therapy has revolutionized the landscape of treatment for hematologic malignancies. This includes multiple myeloma (MM), acute lymphoblastic B-cell leukemia (B-ALL), and non-Hodgkin lymphoma (NHL). This review delves into the pivotal trials of approved autologous CAR-T therapies (auto-CARTs) and new CAR-T constructs that are under investigation to try to help overcome the limitations seen in currently approved CAR-T therapies.

## 2 NHL

### 2.1 Indolent B-cell non-Hodgkin lymphomas

#### 2.1.1 Axi-cel

**Figure 1:**
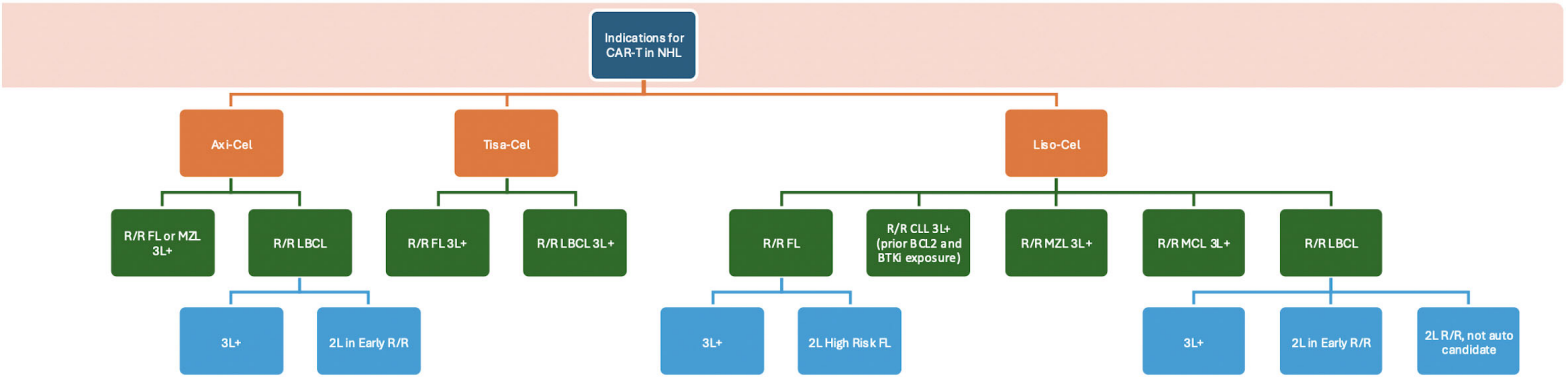
Indications for CAR-T in NHL.

Axicabtagene ciloleucel (axi-cel) is an anti-CD19 CAR-T therapy utilizing a CD28 costimulatory domain that is approved for the use of patients with relapsed or refractory (R/R) indolent lymphoma [follicular lymphoma (FL) grade 1–3a or marginal zone lymphoma (MZL)] who have been exposed to two or more lines of therapy. ZUMA-5 (a phase 2, single-arm, multicenter trial) led to the approval of axi-cel for this indication. The primary end point was overall response rate (ORR). Of the 148 treated patients enrolled, 124 had FL, and 24 had MZL. This included patients with FL who experienced progression of disease within 24 months of initial treatment (POD24) (55%), high tumor bulk (52%), and high-risk Follicular Lymphoma International Prognostic Index (FLIPI) (44%). The median prior lines of therapies were three for both the FL and MZL groups. Bridging therapy was received by 4% of the patients: four patients with FL and two patients with MZL. The bridging therapies were most commonly Rituximab, Ifosfamide, Carboplatin, Etoposide (RICE)- or Bendamustine and Rituximab (BR)-based. The median time from leukapheresis to availability was 17 days. The updated median follow-up was 23.3 months to assess the duration of response (DOR). The ORR at that time for the total patient population was 92% (95% CI 85–96). Complete response (CR) was seen in 76%, and 16% had a partial response (PR). For patients with FL, the ORR was 94% (95% CI 87–98); 79% were in CR, and 15% were in PR. MZL patients had an 83% ORR (95% CI 61–96), with 6% in CR and 17% in PR. At 18 months, progression-free survival (PFS) was estimated at 64.8% (95% CI 54.2–73.5), and overall survival (OS) was 87.4% (95% CI 79.2–92.5). The median time to initial response was 1 month across both the FL and MZL groups (**Figure 1**).

Adverse events (AEs) were common, with 99% of patients having any-grade AEs, and 86% of patients had grade 3 or higher events. The most common grade 3 or higher AE was cytopenias (70%), which consisted mostly of neutropenia. The next most common grade 3 or worse AE was infection (18%).

Cytokine release syndrome (CRS) occurred in 84% of patients and was mostly grade 1 or 2 (75%). Grade 3 or worse CRS occurred in 7% of patients. The median onset of CRS after infusion was 4 days. All CRS events were resolved except one that led to multisystem organ failure and death on day 7. This patient had bulky FL disease according to the Groupe d'Etude des Lymphomes Folliculaires (GELF) criteria.

Neurological events occurred in 59% of patients, with grade 1 or 2 events in 40% of patients and grade 3 or 4 events in 19% of patients. No grade 5 events occurred. The median onset of neurological events was 7 days from infusion, and the median duration was 14 days for patients with FL and 10 days for patients with MZL (1).

The 5-year follow-up from ZUMA-5 showed a durable response to axi-cel with a median DOR of 60.4 months [95% CI 39.7–not estimable (NE)]. The 60-month OS estimate was 69.0% (95% CI 60.8–75.8), and the median OS was not reached (NR) (95% CI NE–NE). The median PFS was 6.2 months (95% CI 34.9–NE), and 50.4% reached the 60-month landmark. Patients with FL had a median PFS of 57.3 months, and patients with MZL had a median PFS of 46.9 months. Rates of PFS were similar among FL patients, including the POD24 cohort. Among patients who had a CR, 58% remained in CR at the time of data cutoff. Also, patients with CR had a median PFS of NR (95% CI 62.2–NE). For patients with PR, the median PFS was 6.9 months (95% CI 4.5–12.4). Of the total patients, 55% were alive without a new anti-cancer therapy. No new safety signals were reported (2).

#### 2.1.2 Real-world data

Initial real-world data regarding axi-cel to treat R/R FL showed overall similar results to those seen in ZUMA-5. Using data available from the Center for International Blood and Marrow Transplant Research (CIBMTR) registry, the outcomes of 151 patients with a median follow-up of 6.2 months were analyzed. The ORR was 93% (95% CI 88–97), and the complete remission rate (CRR) was 84% (95% CI 77–89). The estimated 6-month PFS was 88% (95% CI 81–92), and OS was 96% (95% CI 91–98). Grade 3 or higher CRS occurred in 2% of patients, and Immune Effector Cell Associated Neurotoxicity Syndrome (ICANS) occurred in 13% of patients. The median time for CRS resolution was 5 days, and ICANS was 4 days (3).

#### 2.1.3 Tisa-cel

Tisagenlecleucel (tisa-cel) is an anti-CD19 CAR-T therapy utilizing a 4-1BB costimulatory domain that is approved for the use of patients with R/R FL grades 1–3a who have been exposed to two or more lines of therapy. ELARA (a phase 2, single-arm, multicenter, open-label trial) led to the approval of tisa-cel for R/R FL in the third-line setting. Ninety-eight patients were enrolled in the study, and ninety-seven patients received tisa-cel. High-risk patients were included, with 64% having bulky disease, 63% experiencing POD24, and 60% with a high FLIPI score. They had a median of four prior lines of therapy. Bridging therapy was received by 45%. The median time from patient enrollment to infusion of tisa-cel was 46 days. The primary end point was CRR, and ORR was the secondary end point. At primary analysis (median follow-up 16.59 months), the CRR was 69.1% (95% CI 58.8–78.3), and the ORR was 86.2% (95% CI 77.5–92.4). PFS at 12 months was 67%. The estimated DOR at 9 months for patients with CR was 86.5%. POD24 patients had a CRR of 59% (95% CI 45.7–71.4), and those without had a CRR of 87.9% (95% CI 718–96.6). For the patients who were in CR, the estimated DOR rate at 9 months was 86.5% (95% CI 74–93.1), and the PFS rate estimated at 12 months was 85.5% (95% CI 74.7–92.2). The estimated PFS rate at 12 months for all patients was 67% (95% CI 56–76). The median DOR, PFS, and OS were NR.

AEs were common, with 99% of patients experiencing any-grade AEs. Grade 3 or higher AE was seen in 78.4%. The most common grade 3 or higher AE was neutropenia at 42.3%. Any-grade infection was seen in 18.6%, and 5.2% had grade 3 or higher events.

CRS was seen in 49% of patients, and none of them were grade 3 or higher. The median time of onset and duration of CRS were 4 days. Of the patients with CRS, 57% had bulky disease, and none of them experienced grade 3 or higher CRS.

Any-grade neurological events were seen in 37.1%, with three patients having grade 2 or higher neurological events. ICANS was observed in 4.1%, with one patient having grade 4 ICANS with potential concurrent HHV6. The median onset of serious neurological events was 9 days, and the median time of resolution was 2 days (4).

The 3-year follow-up showed continued durability of response. The ORR was 86% (95% CI 77.5–92.4). The median PFS was 37 months. The POD24 cohort had a 36-month PFS of 50%, and those without POD24 had 59%. The median DOR had not been reached at that time, and in patients who had responses, 64% of them had continued responses at 36 months. No new safety signals were observed (5).

#### 2.1.4 Real-world data

The real-world efficacy of tisa-cel in R/R FL was evaluated using the French DESCAR-T registry, with 129 patients. It showed a similar efficacy and safety profile to that seen in ELARA. The median time from leukapheresis to delivery of tisa-cel was 29 days. The ORR was 98.2%, and the CRR was 85.8%. The 12-month PFS was 62.6%, and the OS rate was 84.9%. The median OS and PFS were NR. Less than 1% had grade 3–4 CRS event and/or grade 3–4 ICANS (6).

#### 2.1.5 Liso-cel

Lisocabtagene maraleucel (liso-cel) is a CD19 CAR-T therapy utilizing a 4-1BB costimulatory domain with 1:1 CAR-positive viable T cells of the CD8 and CD4 components that is approved for R/R FL grades 1–3a in the third-line setting and in the second-line setting in high-risk diseases. TRANSCEND FL (a phase 2, single-arm, open-label, multicohort, multicenter study) evaluated the efficacy and safety of liso-cel in patients with R/R indolent NHL. Patients with FL were assigned to the second-line (2L) cohort or the third-line or more (3L+) cohort. The 2L cohort comprised patients who had received one prior line of therapy and had met the POD24 criteria and/or one or more of the Modified Groupe d'Etude des Lymphomes Folliculaires (mGELF) criteria. These patients would thus receive liso-cel in the second-line setting. Patients who had received three or more lines of therapy were in the 3L+ cohort. Of the 139 patients enrolled in this trial, 25 were in the 2L FL cohort, while 114 were assigned to the 3L+ cohort. Ultimately, 101 of the 3L+ cohort and 23 of the 2L cohort were evaluable. The median time from leukapheresis to availability of liso-cel was 29 days, and the time from leukapheresis to infusion of liso-cel was 49 days. Of the patients who were treated, 56% met the mGELF criteria, 45% met the criteria for POD24, and 53% had high-risk FLIPI. Bridging therapy was received by 38%.

The primary end point was ORR, and CRR and DOR were included in the secondary end points. Patients with 3L+ FL had an ORR of 97% (95% CI 91.6–99.4, p < 0.0001), with a CRR of 94% (95% CI 87.5–97.8, p < 0.0001). The median DOR was NR at a median follow-up of 16.6 months, and the 12-month DOR rate was 82% (95% CI 72.5–88.4). The median PFS at a median follow-up of 17.6 months was NR (95% CI 19–NR), and the 12-month PFS rate was 81% (95% CI 71.4–87.2). The median OS was NR, and the 12-month OS rate was 92% (95% CI 84.8–96.0). The median time for the initial response was 1 month.

Patients with 2L FL had an ORR of 96% (95% CI 78.1–99.9, p < 0.0001). All the patients in this cohort who responded achieved a CR. At a median follow-up of 16.8 months, the median DOR rate was NR (95% CI 19.3–NR), and the 12-month duration rate was 90% (95% CI 64.8–97.4). At a median follow-up of 17.8 months, the median PFS was NR (95% CI 20.2–NR), and the median OS was NR. The 12-month OS rate was 96% (95% CI 72.9–99.4). The median time to initial response was 1 month.

Grade 3 or higher AEs occurred in 75% of 3L+ patients and 61% of 2L patients; 25% of 3L+ patients and 17% of 2L patients experienced serious AEs. The most common grade 3 or higher AE was cytopenia, specifically neutropenia. This was experienced in 58% of 3L+ patients and 52% of 2L patients.

Any-grade CRS was seen in 58% of 3L+ patients and 52% of 2L patients. The median onset of CRS was 6 days, and the median duration was 3 days. Most of the CRS events were grade 1 in nature (42% 3L+, 30% 2L). Grade 3 CRS only occurred in one patient (3L). No grade 4 or 5 CRS events occurred.

Any-grade neurological events occurred in 15% of 3L+ patients and 17% of 2L patients. The median onset of a neurological event was 8.5 days, with a median duration of 3.5 days. Most neurological events were grade 1 (12% 3L, 13% 2L). Three patients had grade 3 neurological events (2% 3L, 4% 2L). No grade 4 or 5 events occurred (7).

At the 2-year follow-up, patients continued to have sustained results. The median study follow-up for 3L+ patients was 30 months, and ORR was 97.1% (95% CI 91.7–99.4). CRR was 94.2% (95% CI 87.8–97.8). The median follow-up for 2L patients was 29.5 months with ORR and CRR of 95.7% (95% CI 78.1–99.9). The 24-month DOR was 74.6% (95% CI 64.8–82.1) in 3L+ patients, PFS was 72.5% (95% CI 62.7–80.1), and OS was 88.2% (95% CI 80.1–93.1). For 2L patients, the 24-month DOR was 86.4% (95% CI 63.4–95.4), PFS was 82.6% (95% CI 60.1–93.1), and OS was 95.7% (95% CI 72.9–99.4). No new safety signs were observed (8).

The efficacy and safety of liso-cel were also explored in chronic lymphocytic leukemia/small lymphocytic leukemia (CLL/SLL). This was explored in Transcend CLL 004, a phase 1/2, single-arm, multicenter, open-label study in the USA. Eligibility criteria included R/R SLL/CLL with prior Bruton’s tyrosine kinase inhibitor (BTKi) treatment failure or deemed BTKi ineligible. Those with high-risk cytogenetics had to have received at least two prior lines of therapy, and patients with standard risk features had to have received at least three prior lines of therapy. The primary end point was CR or remission. The secondary end points included ORR, minimal residual disease (MRD), DOR, PFS, and OS. One hundred thirty-seven patients were enrolled, and 117 received liso-cel infusion. They had a median of five prior lines of therapy, and 83% had any high-risk cytogenetics. The median time from leukapheresis to infusion was 36 days. Bridging therapy was received by 76%. CRR in the primary efficacy analysis set was 18% (95% CI 9–32, p = 0.0006). ORR was 43% (95% CI 29–58, p = 0.39). Peripheral blood undetectable MRD rates were 63% (95% CI 48–77) and 59% (95% CI 44–73) in the marrow. The median time to initial response was 1.2 months, and the median time to first CR or remission was 3 months. The median DOR was 35.3 months (95% CI 11.0–NR), and the median duration of CR or remission was NR. The median PFS was 11.9 months (95% CI 5.7–26.2), and the median OS was 30.3 months (95% CI 11.2–NR). Patients with del(17p), mutated tp53, or both had CRR of 23% (95% CI 10–42) and ORR of 47% (95% CI 28–66).

AEs of grade 3 or higher were experienced in 92% of patients. The most common AE was cytopenia, specifically neutropenia (61%). Grade 3 or higher infections occurred in 17% of patients.

Any-grade CRS occurred in 85% of patients. Most CRS events were grade 1–2 (76%). Grade 3 CRS occurred in 9% of patients, and there were no grade 4 or 5 CRS.

Any-grade neurological event occurred in 45% of patients, with 18% of patients having grade 3 neurological events and one patient with a grade 4 neurological event. No grade 5 neurological events were observed (9).

CRR at the additional 16-month follow-up was 20%, ORR was 44%, and the undetectable marrow MRD rate was 60%. The median DOR was NR (95% CI 12.4–NR) with a median follow-up of 31.7 months (95% CI 21.3–35.5). The DOR rate estimated at 36 months was 61% (95% CI 14.5–NR). No new safety signals were observed (10).

Data from the MZL cohort of TRANSCEND FL were recently released. A 95.5% (95% CI 49.3–73.8, p < 0.0001) ORR was observed; 62.1% had a CR, and 88.6% had maintained a response at 24 months. The 24-month PFS rate was 85.7%, and the OS rate was 90.4%. Any-grade CRS was 76%. Grade 3 CRS was observed in 4% of patients, and no grade 4 or 5 CRS events were reported (11) (**Table 1**).

**Table 1:** Overview of CAR-Ts in Indolent Lymphomas.

Disease	Construct	Response rate (ORR/CRR)	CRS rates	CRS median onset/duration	ICANS rates	ICANS median onset/duration
FL/MZL	Axi-cel	92%/74%	Any grade 84%, grade 3 or higher 7%	4 days/6 days	Any grade 59%, grade 3 or higher 19%	7 days/14 days (FL), 10 days (MZL)
FL	Tisa-cel	86%/69%	Any grade 49%, grade 3 or higher 0%	4 days/4 days	Any grade 4%, grade 3 or higher 1%	9 days/2 days
FL	Liso-cel	3L+ 97%/94%; 2L 96%/100%	2L: any grade 52%, grade 3 or higher 0% 3L+ CRS: any grade 58%, grade 3 or higher ~1%	6 days/3 days	2L: any grade 17%, grade 3 or higher 4% 3L+: any grade 15%, grade 3 or higher 2%	8.5 days/3.5 days
CLL	Liso-cel	44%/20%	Any grade 85%, grade 3 or higher 9%	4 days/6 days	Any grade 45%, grade 3 or higher 18%	7 days/7 days

ORR, overall response rate; CRR, complete remission rate; FL, follicular lymphoma; MZL, marginal zone lymphoma; CLL, chronic lymphocytic leukemia; Axi-cel, axicabtagene ciloleucel; Tisa-cel, tisagenlecleucel; Liso-cel, lisocabtagene maraleucel; CRS, cytokine release syndrome.

### 2.2 Aggressive B-cell lymphoma

#### 2.2.1 Axi-cel

ZUMA-1 (phase 1/2, single-arm, multicenter study) explored the safety and efficacy of axi-cel in patients with R/R large B-cell lymphoma (LBCL) and led to the approval of axi-cel in patients with R/R LBCL after two lines of prior therapy. This study included patients with diffuse large B-cell lymphoma (DLBCL), primary mediastinal B-cell lymphoma (PMBCL), and transformation of FL. Of the 111 patients enrolled in the study, 110 had axi-cel manufactured, and 101 patients ended up receiving the infusion. Bridging therapy was not allowed after leukapheresis or before receiving axi-cel. The median time from leukapheresis to availability of axi-cel was 17 days. The primary end point was ORR. The secondary end points included DOR, PFS, and OS. The median time to response was 1 month, and the median DOR was 8.1 months (95% CI 3.3–NE). At the initial follow-up of 6 months, the ORR was 82% (95% CI 73–89), and the CRR was 54%. An updated analysis occurred at a minimum follow-up of 1 year to assess the DOR. The ORR was 82% with a CRR of 58%. At the time of data cutoff, 42% continued to have a response, which included 40% with CR. The median DOR was 11.1 months (95% CI 3.9–NE). The median duration of PFS at 6 months was 5.8 months (95% CI 3.3–NE); at 12 months, it was 44% (95% CI 34–53); and at 18 months, it was 52% (95% CI 41–62). At data cutoff, 56% of patients were alive.

All patients had any-grade AEs. Grade 3 or higher AEs were experienced by 95% of patients. The most common grade AE was fever (85%). The next most common any-grade AEs were neutropenia (84%) and anemia (66%). The majority of grade 3 or higher AE was neutropenia (78%). The next most common grade 3 or higher AEs were anemia (43%) and thrombocytopenia (38%).

CRS was observed in 93% of patients. The majority of CRS events were low grade, with 37% having grade 1 and 44% having grade 2 in nature. Grade 3 CRS accounted for 9% of cases, grade 4 accounted for 3%, and grade 5 accounted for 1%. The median onset of CRS was 2 days after infusion, and the median time to resolution was 8 days.

Neurological events were observed in 64% of patients, with 28% having grade 3 or higher. The median onset of neurological events was 5 days after infusion, and the median resolution was 17 days after infusion (12).

The 5-year follow-up of ZUMA-1 showed sustained DOR of axi-cel in patients with R/R LBCL. The median follow-up was 63.1 months after axi-cel infusion. The ORR was 83% (95% CI 74–90), and the CR was 58%. The median DOR was 11.1 months (95% CI 4.2–51.3), the median duration of CR was 62.2 months, and the median duration of PR was 1.9 months. No new safety events were observed (13).

ZUMA-7 (phase 3, open-label, multicenter, randomized trial) evaluated axi-cel as a second-line therapy in patients with early R/R disease. Patients were eligible for the trial if they were refractory to the first line of therapy or had relapsed from their disease after CR at no more than 12 months after completing the first line of therapy. Patients were assigned in a 1:1 ratio randomly to receive axi-cel versus standard of care (SOC) [chemoimmunotherapy followed by autologous stem cell transplant (auto-SCT) for patients with CR or PR]. Randomization was stratified by response to a first-line therapy (refractory versus relapsed disease) and second-line age-adjusted International Prognostic Index (IPI) factor versus two or three risk factors (which would indicate a high-risk disease). Only glucocorticoids were allowed as optional bridging therapy for the axi-cel cohort. Crossover between treatment groups was not planned. However, those who did not respond to SOC could receive cellular immunotherapy outside of the protocol. The primary end point for this trial was event-free survival (EFS). The secondary end points included response, OS, and PFS. One hundred eighty patients were assigned to the axi-cel group and 179 patients to the SOC group. Of the patients in the axi-cel group, 170 received axi-cel. The median time from leukapheresis to availability was 13 days. Of the patients who were in the SOC arm, 94% received platinum-based salvage chemotherapy, and 36% received high-dose chemotherapy and underwent auto-SCT.

The median EFS was 8.3 months (95% CI 4.5–15.8) in the axi-cel arm and 2 months (95% CI 1.6–2.8) in the SOC arm. The 24-month estimated EFS was 41% (95% CI 33–48) in the axi-cel arm and 16% (95% CI 11–22) in the SOC arm. The EFS curves demonstrated that axi-cel was superior to SOC (95% CI 0.31–0.51, p < 0.001). CR was seen in 65% of the axi-cel group and 32% of the SOC group. At the interim analysis, the median OS was NR in the axi-cel group and 35.1 months in the SOC group (95% CI 0.53–1.01, p = 0.054). The estimated OS at 2 years was 61% in the axi-cel group and 52% in the SOC group. The PFS was 14.7 months in the axi-cel group (95% CI 5.4–NE) and 3.7 months in the SOC group (95% CI 0.37–0.65). The axi-cel group had an estimated PFS at 24 months of 46% (95% CI 38–53), while for the SOC group, it was 27% (95% CI 20–35). Sensitivity analysis of OS was completed to address potential confounding effects of treatment crossover. This showed an improvement of OS with axi-cel (95% CI 0.42–0.81).

All patients had reported at least one AE of any grade. Of the patients who received axi-cel, 91% had a grade 3 or higher, and 83% of patients who received SOC had a grade 3 or higher. The majority of grade 3 or higher AEs were neutropenia, with 69% of axi-cel patients and 41% of SOC patients. Any-grade infection was seen in 50% of axi-cel patients and 46% of SOC patients. Grade 3 or higher infections occurred in 14% of axi-cel patients and 11% of SOC patients.

CRS was reported in 92% of axi-cel patients. Grade 3 or higher CRS occurred in 6% of patients. No deaths related to CRS. The median time to onset of CRS was 3 days after infusion, and the median duration was 7 days.

Neurological events were reported in 60% of axi-cel patients and 20% of SOC patients. Grade 3 or higher neurological events were reported in 21% of axi-cel patients and 1% of SOC patients. The median time to onset of neurological events was 7 days for the axi-cel group, while in the SOC group, it was 23 days. The median duration of neurological events for the axi-cel group was 9 days, and it was 23 days for the SOC group (14).

A 5-year follow-up was evaluated to determine the long-term outcomes of early R/R LBCL patients who received axi-cel versus SOC. Primary analysis showed OS improvement with axi-cel in comparison to SOC (95% CI 0.54–0.98, p=0.03). The estimated OS at 2 years was 54.6% (95% CI 47–61.6) in the axi-cel group and 46.0% (95% CI 38.4–53.2) in the SOC group. The median OS was NR in the axi-cel group (95% CI 28.6–NE) and 31.1 months (95% CI 17.1–NE) in the SOC group. Sensitivity analysis was conducted to evaluate the potential confounding effect of treatment crossover on OS in the SOC arm, which demonstrated a greater OS benefit with axi-cel. The median PFS was 14.7 months with axi-cel (95% CI 5.4–43.5) and 3.7 months (95% CI 2.9–5.3) with SOC. The estimated PFS at 4 years for axi-cel was 41.8% (95% CI 34.1–49.2) and 24.4% (95% CI 17.2–32.2) for SOC. The median EFS was 10.8 months (95% CI 5–25.5) for axi-cel and 2.3 months (95% CI 1.7–3.1) for SOC. No new safety signals were observed (15).

#### 2.2.2 Real-world data

The real-world data of axi-cel as a second line for patients with early R/R LBCL were explored using data from the CIBMTR, with a total of 446 patients. At a median follow-up of 12 months, ORR was 79% (95% CI 75–82), and CRR was 64% (95% CI 60–69). The DOR was 66% (95% CI 59–71), PFS was 53% (95% CI 48–58), EFS was 53% (95% CI 48–58), and OS was 71% (95% CI 66–76); 87% had any-grade CRS, with 5% having grade 3 or higher. ICANS was seen in 50%, and grade 3 or higher was seen in 22%. Outcomes and safety signals were similar to those seen in ZUMA-7 (16).

#### 2.2.3 Tisa-cel

Tisa-cel was evaluated in patients with R/R DLBCL in the trial JULIET (phase 2, single-group, open-label, multicenter trial). To be eligible for this trial, patients had to have received two prior lines of therapy (which included rituximab and an anthracycline). Patients either had to have a relapse after an auto-SCT or were ineligible for auto-SCT. Patients with DLBCL transformed from FL and high-grade B-cell lymphoma (HGBCL) with double or triple hit were included. Patients with active central nervous system (CNS) involvement of DLBCL were excluded from this trial. The primary end point was the best ORR. The secondary end points included DOR and OS. One hundred sixty-five patients were enrolled, and 111 received tisa-cel infusion. The median time from enrollment to tisa-cel infusion was 54 days. Bridging therapy was received by 92%. The best ORR for patients who had 3 months or more of follow-up or had discontinued participation in the study before 3 months was 52% (95% CI 41–62); 40% of patients had a CR, and 12% had a PR. At 6 months, the overall response was 33%, and CR was 29%. The median DOR was NR (95% CI 10–NR). Of the patients who had CR, it was estimated that 79% (95% CI 60–89) remained relapse-free at 12 months after having a response. The median PFS was NR for patients with CR. The median OS of patients who received tisa-cel was 12 months (95% CI 7–NR).

The most common grade AE was CRS (58%). This was followed by pyrexia (35%), neutropenia (34%), thrombocytopenia (33%), leukopenia (33%), and diarrhea (32%). Grade 3 or 4 AEs included CRS with 22% of patients, cytopenias not resolved by day 28 (32%), and infections (20%). The median time from onset of CRS from infusion was 3 days, and the median duration was 7 days.

Any-grade neurological events occurred in 21% of patients within 8 weeks after infusion. The median time of onset was 6 days from infusion, and the median duration was 14 days; 12% had grade 3 or 4 events (17).

Long-term follow-up was completed to evaluate the long-term response to tisa-cel in R/R DLBCL. The median follow-up was 40.3 months, with a maximum follow-up of 52.6 months. The ORR was 53.0% (95% CI 43.5–63.4); 39% had CR as the best overall response, and the median DOR was NE (95% CI 10.0–NE). The median PFS was 2.9 months (95% CI 2.3–5.2), and for patients who had a CR at 3 or 6 months, the median PFS was NR. The median OS was 11.1 months (95% CI 6.6–23.9), and the median OS for patients who had CR at 3 or 6 months or as the best overall response was NR. No new safety signals were noted (18).

Tisa-cel was compared to SOC chemotherapy and auto-SCT in the second-line setting for refractory LBCL and those who progressed within 12 months from a first-line therapy. This was evaluated in BELINDA, a phase 3, international, randomized trial. Crossover was allowed from the tisa-cel group to the SOC arm. The primary end point was EFS. The median EFS in both arms was 3 months (95% CI 0.82–1.4, p = 0.61). Ultimately, it was found that tisa-cel was not better than SOC (19).

#### 2.2.4 Real-world data

Real-world experience with tisa-cel in R/R DLBCL or HGBCL after two or more lines of therapy was evaluated using CIBMTR data, with 1,159 patients. At a median follow-up of 23.2 months, the ORR was 59.5%, the CRR was 44.5%, PFS was 28.4%, OS was 43.6 months, and the ongoing response was 52.6%. Grade 3 or higher CRS was seen in 6%, and ICANS was seen in 7.4%. These efficacy findings were similar to those seen in JULIET and better safety outcomes (20).

#### 2.2.5 Liso-cel

The safety and efficacy of liso-cel in R/R LBCL in the third-line setting were explored in TRANSCEND NHL 001 (multicohort, multicenter, seamless design study). Patients were eligible if they had R/R DLBCL and had received two or more lines of prior treatment (including prior chemoimmunotherapy containing anti-CD20 and anthracycline). They included HGBCL (double-hit or triple-hit lymphoma), PMBCL, or FL grade 3b. Patients with secondary CNS involvement were eligible as well. The median time from leukapheresis to availability of liso-cel for shipment to the study site was 24 days, and the median time to infusion was 37 days. The primary end point was ORR and included the incidence of AEs and the probability of dose-limiting toxicities. The secondary end points included CRR, PFS, OS, and DOR. Three hundred forty-four patients were enrolled in the study and underwent leukapheresis. Two hundred sixty-nine patients received CAR-T cells. The median prior lines of therapy were three. The total number of patients with secondary CNS involvement was 7%. Bridging therapy was received by 59% of patients at the investigator’s discretion, and it did not result in a lower tumor burden in most patients. In the efficacy evaluable set, 73% (95% CI 66.8–78.0, p < 0.0001) of patients had an objective response, and 53% (95% CI 46.8–59.4, p < 0.0001) had a CR. The median time to first CR or PR was 1 month. The 1-year estimated DOR rate was 55% (95% CI 11.2–16.7) for the total patient population and 65% (95% CI 56.2–72.8) for those who had a CR. The median PFS was 6.8 months (95% CI 3.3–14.1) at a median follow-up of 12.3 months (95% CI 12–17.5). Those who had a CR median PFS were NR. For the total patient population, the 1-year PFS was 44% (95% CI 37.3–50.7), and it was 65% (95% CI 56.1–72.7) for patients who had CR. The median OS for those in CR was NR, and the OS was 86% (95% CI 78.2–90.5) at 1 year. Of the seven patients who had secondary CNS disease, three had CR. Durable responses were seen across all LBCL subtypes, which included chemotherapy-refractory disease and high tumor burden.

The most common all-grade AEs were neutropenia (63%), anemia (48%), and fatigue (44%). The most common grade 3 or higher AEs were neutropenia (60%), anemia (37%), and thrombocytopenia (27%).

CRS of any grade occurred in 42% of patients, and the median onset was 5 days. Grade 3 or higher CRS was seen in 2% of patients. None of the patients passed away due to CRS.

Any-grade neurological events were reported in 30% of patients and had a median time of onset of 9 days. Grade 3 or higher neurological events were observed in 10%. Of the seven patients with secondary CNS disease, two (29%) had grade 3 neurological events (21).

The 2-year follow-up showed a durable response to liso-cel. In the efficacy evaluable set, the median duration was 23.1 months (95% CI 8.6–NR), with a median follow-up of 23 months (95% CI 22.8–23.1). The median DOR of patients who had CR was 26.1 months (95% CI 23.1–NR). The ORR was 73% (95% CI 66.9–78.1), and 53% best response was CR (95% CI 46.6–59.2). The median PFS was 6.8 months (95% CI 3.3–12.7) with a median follow-up of 23.9 months (95% CI 23.7–24). The median PFS for those who had CR was 27.3 months (95% CI 24–NR). The median OS was 27.3 months (95% CI 16.2–45.6) with a median follow-up of 29.3 months (95% CI 26.2–30.4). The median OS for those who had CR was 48.5 months (95% CI 45.2–NR). No new safety signals were observed. At the 5-year follow-up, the estimated OS rate was 38.1% (95% CI 31.6–44.7) (22).

Liso-cel was investigated as a second-line therapy in patients with R/R LBCL who are ineligible for auto-SCT in the PILOT trial (phase 2, open-label study). Patients were eligible if they received a first-line therapy that contained an anthracycline and a CD20-targeted agent. They also must not have intended to undergo auto-SCT. HGBCL (double hit or triple hit), transformed follicular to DLBCL, and FL grade 3b were included. The primary end point was the ORR. The secondary end points included CRR, DOR, PFS, and EFS. Bridging therapy was allowed. Seventy-four patients underwent leukapheresis, and 61 patients received liso-cel. The median time from leukapheresis to infusion was approximately 35 days. Bridging therapy was received by 52%. Overall response was seen in 80% (95% CI 68–89, p < 0.0001), and 54% (95% CI 41–67) had CR. The median PFS in all patients at a median follow-up of 13 months was 9 months (95% CI 12–NR). The median EFS in all patients at a median follow-up of 16.4 months was 7.2 months (95% CI 3.22–22.6). The median OS at a median follow-up of 17.6 months was NR (95% CI 17.28–NR). For those who had CR, the median PFS was 22.6 months (95% CI 12.98–NR), and the median OS was NR (95% CI NR–NR). The median DOR was 21.7 months (95% CI 12.09–NR). The median time to first response was 1 month, and the median time to first CR was 1 month.

The most common all-grade AEs were neutropenia (50%), and the most common grade 3 or higher AEs were neutropenia (48%), leukopenia (21%), and thrombocytopenia (20%); 7% had grade 3 or higher infections.

All-grade CRS occurred in 38% of patients, with 36% having grades 1–2 and 2% having grade 3. No grade 4 or 5 CRS occurred.

Any-grade neurological events were observed in 31%, with grade 1–2 in 26% and grade 3 in 5%. There were no grade 4 or 5 neurological events (23).

Later, the final results of the PILOT study were reported. The median follow-up was 18.2 months. The ORR was 80%, with 54% achieving CR. The median DOR was 23.3 months (95% CI 6.2–NR), the median PFS was 9 months (95% CI 4.2–NR), and the median OS was NR (95% CI 16.3–NR). The 18-month PFS rate was 43% (95% CI 30–55), and the OS rate was 59% (95% CI 45–70). No new safety signals were reported (24).

Liso-cel was also investigated in the second-line setting for patients with early (within 12 months after initial therapy) R/R LBCL who were transplant-eligible in comparison to SOC salvage immunochemotherapy followed by auto-SCT as consolidation in the pivotal study TRANSFORM. This study was a phase 3, global, open-label, randomized study. It included PMBCL, FL grade 3b, transformed LBCL from indolent NHL, HGBCL with myc and BCL2 or BCL6 or both rearrangements, and T-cell histiocyte-rich LBCL. It also included patients with secondary CNS lymphoma. Patients were able to receive one cycle of bridging therapy during liso-cel manufacturing. Crossover from SOC to liso-cel was allowed. The primary end point was EFS. The secondary end points included CRR, PFS, OS, DOR, and ORR. One hundred eighty-four patients were enrolled, and 93 were designated to the liso-cel group and 92 to the SOC group. Of those assigned to the liso-cel arm, 89 received liso-cel infusion. Bridging therapy was received by 63% in the liso-cel arm. The median time from leukapheresis to liso-cel availability was 26 days, and the time from leukapheresis to infusion was 36 days. Improvement of EFS was seen in all prespecified subgroups when comparing liso-cel to SOC. CRR was 66% (95% CI 56–76) in the liso-cel group and 39% in the SOC group (95% CI 29–50, p < 0.0001). Those who had a CR with liso-cel had a longer median DOR in comparison to SOC, with NR (95% CI 6.8–NR) and 14.5 months (95% CI 4.7–NR), respectively. The ORR with liso-cel was 86% (95% CI 77–92), and that with SOC was 48% (95% CI 37–59). PFS and OS in liso-cel group were improved in comparison to SOC (95% CI 0.25–0.66, p = 0.0001; 0.26–1, p = 0.026, respectively).

The most common grade 3 or higher AEs with liso-cel were neutropenia (80%), anemia (49%), and thrombocytopenia (49%). Prolonged cytopenia was seen in 43% of patients in the liso-cel group, and 73% improved to grade 2 or better within 2 months from onset.

Any-grade CRS was seen in 49% of patients. Most CRS events were grade 1 or 2 in nature, with 37% and 11%, respectively. Grade 3 CRS was seen in only 1% of patients, and no grade 4 or 5 was seen. The median time from infusion to onset of CRS was 5 days, and resolution was 4 days.

Of the patients in the liso-cel group, 12% had any-grade neurological event. Grade 1 neurological events were seen in 5%, grade 2 in 2%, and grade 3 in 4%. No grade 4 or 5 neurological events were seen (25).

A 3-year follow-up from TRANSFORM was completed. The median follow-up was 33.9 months. The median EFS was 29.5 months for the liso-cel group (95% CI 9.5–NR) and 2.4 months for the SOC group (95% CI 0.259–0.542). The median OS for liso-cel and SOC were NR. The 36-month OS rate was 63% for liso-cel and 52% for SOC. No new safety signals were seen (26).

#### 2.2.6 Real-world data

The real-world data of liso-cel in treating R/R LBCL were evaluated retrospectively across seven US medical centers with 101 patients. The median lines of prior therapies were three; 60% of patients received bridging therapy. After liso-cel infusion, the ORR was 66% at day 90. At a median follow-up of 15.5 months, 12-month PFS and OS were 55% and 68%, respectively. CRS of any grade was seen in 49%, with 3% having grade 3 or higher. Any-grade ICANS was seen in 26%, with 10% having grade 3 or higher. This study showed similar efficacy and safety findings to prior pivotal trial results (27) (**Table 2**).

**Table 2:** Overview of CAR-Ts in LBCL.

Construct	Indication	Response rate (ORR/CRR)	CRS rates	CRS median onset/duration	ICANS rates	ICANS median onset/median duration
Axi-cel	R/R LBCL	82%/54%	Any grade 93%, grade 3 or higher 13%	2 days/8 days	Any grade 64%, grade 3 or higher 28%	5 days/17 days
	Early R/R LBCL	83%/65%	Any grade 92%, grade 3 or higher 6%	3 days/7 days	Any grade 60%, grade 3 or higher 21%	7 days/9 days
Tisa-cel	R/R LBCL	34%/40%	Any grade 58%, grade 3 or higher 22%	3 days/7 days	Any grade 21% grade 3 or higher 12%	6 days/14 days
Liso-cel	R/R LBCL	73%/53%	Any grade 42%, grade 3 or higher 2%	5 days/5 days	Any grade 30%, grade 3 or higher 10%	9 days/11 days
	Early R/R LBCL	86%/66%	Any grade 49%, grade 3 or higher 1%	5 days/4 days	Any grade 12%, grade 3 or higher 4%	11 days/6 days
	R/R LBCL, not auto candidate	80%/54%	Any grade 38%, grade 3 or higher 2%	4 days/4 days	Any grade 31%, grade 3 or higher 5%	7 days/6 days

ORR, overall response rate; CRR, complete remission rate; CRS, cytokine release syndrome; Axi-cel, axicabtagene ciloleucel; Tisa-cel, tisagenlecleucel; Liso-cel, lisocabtagene maraleucel; R/R LBCL, relapsed or refractory large B-cell lymphoma.

### 2.3 Mantle cell lymphoma

#### 2.3.1 Brexu-cel

Brexucabtagene autoleucel (brexu-cel) is approved for R/R mantle cell lymphoma (MCL) in the third-line setting. ZUMA-2 (phase 2, single-group, multicenter, open-label study) led to the approval of brexu-cel for this indication. Brexu-cel is a CD19-targeting CAR-T with a CD28 costimulatory domain and involves a process to remove leukemic phase cells and prevent premature T-cell exhaustion during manufacturing. Patients were eligible to enroll if they had R/R MCL up to five prior regimens. Prior therapies must have included anthracycline- or bendamustine-containing chemotherapy, an anti-CD20 monoclonal antibody, and BTKi. The primary end point was ORR. The secondary end points included DOR, PFS, and OS. Seventy-four patients were enrolled, and 68 patients received brexu-cel infusion. The median time from leukapheresis to delivery of brexu-cel to the study site was 16 days. Most patients received at least three prior lines of therapy, and 88% were refractory to BTKi therapy. Bridging therapy was received by 37%. Of all enrolled patients, 85% had an objective response, and 59% had a CR. The objective response was similar among all subgroups, which included patients with high-risk features. The median time to initial response was 1 month, and the median time to CR was 3 months. The estimated 12-month PFS was 61%, and the estimated 12-month OS was 83%. PFS at 6 months was consistent among subgroups of patients.

All treated patients had an AE of any grade. Grade 3 or higher events occurred in 99% of patients. The most common grade 3 or higher AEs were cytopenias (94%). The next most common grade 3 or higher AEs were infections (32%). The majority of grade 3 or higher cytopenias were neutropenia (85%), thrombocytopenia (51%), and anemia (50%).

CRS was observed in 91% of patients. The majority of CRS cases were grade 1 or 2 in nature (76%). Grade 3 or higher CRS was seen in 15% of patients. The median time after receiving brexu-cel to any-grade CRS was 2 days, and for grade 3 or higher CRS, it was 4 days. These CRS events resolved at a median of 11 days.

Neurological events occurred in 63% of patients. Grade 1 or 2 neurological events were seen in 32% of patients, and grade 3 or higher events in 31% of patients. The median time to onset of any-grade neurological event was 7 days, and grade 3 neurological event was 8 days. The median duration was 12 days (28).

A 3-year follow-up of ZUMA-2 was later reported. The median follow-up was 35.6 months. The ORR was 81% (95% CI 81.8–96.7), and CR was 68% (95% CI 55.2–78.5). The median DOR was 28.2 months (95% CI 13.5–47.1), the median PFS was 25.8 months (95% CI 9.6–47.6), and OS was 46.6 months (95% CI 24.9–NE). The ORRs were consistent across the prespecified subgroups, which included those with high-risk characteristics. No new safety signals were seen (29).

#### 2.3.2 Real-world data

The real-world effectiveness and safety of brexu-cel were examined in a prospective study of patients with R/R MCL. This study used CIBMTR data, with 476 patients. At a median follow-up of 13.5 months, an ORR of 91% was seen. CRR was 82%, and the 1-year OS and PFS were 76% and 63%, respectively. Those who had a CR or PR as the best response were 79% (95% CI 74–82) at 6 months, and those who had an ongoing response were 66% at 12 months. At 1 year, the non-relapse mortality was 8%. Any-grade CRS was seen in 89%, with 11% having grade 3 or higher. Any-grade ICANS was seen in 62%, with 30% having grade 3 or higher. Thus, this real-world study showed similar safety and efficacy results as seen in ZUMA-2 (30).

#### 2.3.3 Liso-cel

TRANSCEND-MCL was an open-label, multicenter study that explored the safety and efficacy of liso-cel in patients with R/R MCL after two or more lines of prior therapy who were positron emission tomography (PET)-positive. This included a BTKi, an alkylating agent, and a CD20-targeted agent. Patients with secondary CNS lymphoma were included, as well as those who had prior auto-SCT or allo-SCT. Bridging therapy was allowed. Primary end points were AEs, probability of dose-limiting toxicity, and ORR. The secondary end points included CRR, DOR, PFS, and OS. One hundred four patients were enrolled, and 92 ended up receiving liso-cel; 31% had blastoid morphology, 23% tp53 mutation, and 8% secondary CNS disease. The median time from leukapheresis to availability to liso-cel was 24.5 days, and that to infusion was 39 days. The median number of prior lines of therapy was three. Bridging therapy was received by 66% of patients. ORR in the primary analysis set was 86.5% (95% CI 76.5–93.3, p < 0.0001). CRR was 74.3% (95% CI 62.8–83.8, p < 0.0001). The median time to first CR or PR was 1 month. At a median follow-up of 22.8 months (95% CI 16.7–23.0), the median DOR was 15.7 months (95% CI 6.2–24.0). At a median follow-up of 23.5 months (95% CI 17.7–23.8), the median PFS was 15.3 months. At a median follow-up of 24 months (95% CI 23.7–24.2), the median OS was 18.2 months (95% CI 12.9–36.3). For patients who had achieved CR, the median OS was 36.3 months (95% CI 15.7–NR).

Grade 3 or higher AEs were observed in 86% of patients, while 100% had any-grade AEs. The most common AEs were cytopenias. The most common cytopenia at any grade and grade 3 or higher was neutropenia at 59% and 56%, respectively. Grade 3 or higher infections was seen in 15% of patients had grade 3 or higher infections.

Any-grade CRS was seen in 61% of patients. The majority of CRS events were grade 1 or 2, as seen in 60% of patients. There were no grade 3 or 5 CRS events. There was only one grade 4 CRS event. The median time to onset of CRS was 4 days, and the resolution of CRS was 4 days as well.

Any-grade neurological event was seen in 31%. The majority were grade 1 or 2 events seen in 22% of patients; 8% having grade 3 neurological events, 1% having grade 4 event, and no grade 5 events. The median time to onset was 8 days, and the median time to resolution was 5 days (31).

At final analysis, with a median follow-up of 16.1 months, continued durable responses were seen. The median DOR was 15.7 months, the median PFS was 15.3 months, and the median OS was 18.2 months. No new safety signals were seen (32).

## 3 B-cell acute lymphoblastic leukemia

### 3.1 Tisa-cel

In the pivotal study ELIANA, a phase 2, single-cohort, multicenter, global study, tisa-cel was investigated in treated CD19+ R/R B-ALL in the children and young adult population. Ages eligible for the trial were 3 to 21 years at diagnosis. Those who had received anti-CD19 therapy previously were excluded. The primary end point was an ORR higher than 20%. The secondary end points included CRR, EFS, and OS. Ninety-two patients were enrolled in the study, and 75 patients received tisa-cel infusion. The median time from enrollment to infusion was 45 days. The median prior lines of therapy were three; 61% had previously undergone Allogeneic hematopoietic stem cell transplant (allo-HSCT). At the interim analysis, the primary end point was met. ORR was 82% (95% CI 69–91, p < 0.001). At the later follow-up of at least 3 months, the ORR was 81% (95% CI 71–89), 60% had CR, and 21% had CR with incomplete recovery (CRi). Those who had CR were negative for MRD, and 95% of these patients were MRD-negative by day 28. ORR was similar among the total population and subgroups. For those with CR, the median DOR was NR. At 6 months, the EFS rate was 73% (95% CI 60–82), and at 12 months, it was 50% (95% CI 35–64). At 6 months, the rate of OS was 90% (95% CI 81–95), and at 12 months, it was 76% (95% CI 63–86).

AE related to tisa-cel was seen in 95%. CRS was the most common non-hematologic AEs of any grade at 77%. Febrile neutropenia was seen in 35% of patients; 73% had a grade 3 or 4 AE related to tisa-cel. Any-grade infection occurred in 43% of patients, 21% were grade 3 in nature, and 3% were grade 4.

The median time to CRS from infusion was 3 days, and the median duration was 8 days; 47% of patients with CRS were admitted to the intensive care unit (ICU) for CRS management; 21% had grade 3 CRS, and 25% had grade 4 CRS.

Neurological events were observed in 40% of patients within 8 weeks after infusion; 13% having grade 3 neurological events were seen in 13%, with no grade 4 events. Within 10 days, 50% of these events had resolved, and 75% had resolved within 18 days (33).

The 3-year follow-up with a median follow-up of 38.8 months showed an ORR of 82%. At 24 months, the median EFS and the median OS were NR. At 3 years, the OS was 63% (95% CI 51–73), and EFS was 44% (95% CI 31–57). No new safety signals were seen (34).

#### 3.1.1 Real-world data

Real-world data regarding tisa-cel in R/R pediatric and young adults with B-ALL were evaluated using CIBMTR data, with 768 patients. At a median follow-up of 32.1 months, the best overall response (CR/CRi) seen was 86%. At 12 months, the OS and relapse-free survival were 79.4% and 61.8%, respectively. At 24 months, it was 63.8% and 50.3%, respectively. Any-grade CRS was seen at 57.5%, with 16.4 grade 3 or higher. Any-grade ICANS was seen in 22.2%, with 9% having grade 3 or higher. Overall, similar efficacy was seen with an improved safety profile compared to that seen in ELIANA (35).

### 3.2 Brexu-cel

ZUMA-3 investigated the efficacy and safety of KTE-X19, otherwise known as brexu-cel, in adult patients with R/R B-ALL. This was a phase 2, single-arm, open-label study. Patients had to have relapse within 12 months of remission or less, R/R disease after at least two prior lines of therapy, or R/R after allo-SCT. Patients were still eligible even if they had previously received blinatumomab. The primary end point was overall CRR or Complete Remission with Incomplete Count Recovery (CRRi). The secondary end points included MRD negativity rate, DOR, relapse-free survival, and OS. Seventy-one patients were enrolled, 92% had brexu-cel manufactured successfully, and 77% received the infusion. The median time from leukapheresis to release of brexu-cel was 13 days for those in the USA and 14.5 days for those in Europe. Three or more lines of therapy were received by 47%, and 45% had previously received blinatumomab. Bridging chemotherapy was given to 93%. CR or CR with incomplete hematologic recovery (CRi) was experienced by 71% (95% CI 57–82, p < 0.0001), and 56% had CR. These results were similar among subgroups. The median time to first CR or CRi was 1.1 months. MRD negativity was seen in 76% of treated patients (p < 0.0001), and of those who responded, 97% had MRD negativity. Allo-SCT was received by 18% of patients after brexu-cel infusion; the median time from infusion to allo-SCT was 98 days. The median DOR was 12.8 months (95% CI 8.7–NE and 9.4–NE, respectively). The median relapse-free survival in all treated patients was 11.6 months (95% CI 2.7–15.5) and 14.2 months (95% CI 11.6–NE) for those who responded. At 12 months, the OS rate was 71% (95% CI 57–82). In all treated patients, the median OS was 18.2 months (95% CI 15.9–NE), and it was NR in those who responded.

All treated patients had an AE. The most common grade 3 or higher AE was anemia at 49%. The second most common was pyrexia at 36%. Grade 3 or higher cytopenia was seen in 76% of patients. Severe AEs were seen in 75% of patients; 25% of patients had grade 3 or higher infections. CRS was seen in 89%, with 24% were grade 3 or 4 in nature. The median time to onset of CRS from infusion was 5 days, and the median duration was approximately 8 days.

Neurological events were experienced by 60% of patients. Grade 3 or higher neurological events were seen in 25% of patients, with one grade 5 event (brain herniation) (36).

A 3-year follow-up of patients from ZUMA-3 was completed. The median follow-up for all patients was 41.6 months, with a median OS of 25.6 months (95% CI 1.2–47). Those who responded had a median OS of 38.9 months (95% CI 25.4–NE). No new safety signals were observed (37).

#### 3.2.1 Real-world data

The real-world outcomes of brexu-cel in adult patients with R/R B-ALL were evaluated using CIBMTR data, with 138 patients. At day 100 post-brexu-cel infusion, the overall CRR/CRRi was 76%, with 70% still in remission at 6 months after their initial response (95% CI 55–80). At 6 months, the OS was 78% (95% CI 69–84), and relapse-free survival was 53% (95% CI 42–62). Of those who passed away, the primary causes of death were either primary disease (41%) or infection (22%). Any-grade CRS was seen in 81% and ICANS in 48%. Grade 3 or higher CRS was seen in 9% and ICANS in 24%. These safety and efficacy findings were similar to those seen with ZUMA-3 (38).

### 3.3 Obe-cel

Obecabtagene autoleucel (obe-cel) is an anti-CD19 CAR-T therapy utilizing a 4-1BB costimulatory domain with a low-affinity, fast off-rate CD19 binding domain with a lower dose infusion at day 1, followed by the main infusion on day 10. Its efficacy and safety in adult patients with R/R B-ALL were evaluated in the clinical trial FELIX. This trial was a phase 1/2, multicenter study. The phase 2 portion of the trial had a main cohort, cohort 2A, for patients with morphologic disease at enrollment. It also had exploratory cohorts: cohort 2B for patients who were MRD-positive and cohort 2C for patients who had isolated extramedullary disease. The primary end point was overall remission for those in cohort 2A, and a key secondary end point was CR. The secondary end points also included DOR, EFS, MRD negativity, OS, and PFS. One hundred fifty-three patients were enrolled in the trial, and 127 patients received at least one obe-cel infusion and were evaluable. The median time from leukapheresis to release of obe-cel was 21 days. Of the patients in cohort 2A, the median prior lines of therapy were two. Of the total patients enrolled, 44.1% had previously undergone allo-HCT, 41.7% had prior exposure to blinatumomab, and 28.3% were Ph-positive. Bridging therapy was received by 92.9%. The planned two doses of obe-cel were received by 94.5%. For cohort 2A, the median follow-up was 20.3 months, and overall remission for those who received at least one infusion of obe-cel was 77% (95% CI 67–85). CR was seen in 55% of patients (95% CI 45–66), and 21% had CRi (95% CI 8.2–NE). Of all patients who received at least one infusion of obe-cel, the median follow-up was 21.5 months, and the overall response was 78% (95% CI 70–85). The median DOR was 21.2 months (95% CI 11.6–NE). The median OS was 15.6 months (95% CI 12.9–NE).

Febrile neutropenia was seen in 24.4% of patients. The median time to neutrophil recovery from infusion was 21 days (95% CI 15–27).

CRS was seen in 68.5% of patients, with grade 3 or higher events in 2.4%. The median time to onset of CRS after receiving obe-cel infusion was 8 days, and the median duration was 5 days.

ICANS was seen in 22.8% of patients, with grade 3 or higher seen in 7.1% of patients. The median onset of ICANS after obe-cel infusion was 12 days, and the median duration was 8 days. Of the patients who had grade 3 or higher ICANS, 56% had more than 75% blasts in their bone marrow prior to lymphodepletion (39).

## 4 Multiple myeloma

### 4.1 Ide-cel

KarMMa is a phase 2 study that evaluated the efficacy and safety of idecabtagene vicleucel (ide-cel), a B-cell maturation antigen (BCMA)-directed therapy utilizing a 4-1BB costimulatory domain, in patients with R/R MM. Patients had to have received at least three prior regimens for MM. This included an immunomodulatory agent, a proteasome inhibitor, and an anti-CD38 antibody. Refractory disease was defined according to the International Myeloma Working Group (IMWG) criteria. Bridging therapy was allowed while manufacturing occurred.

Overall response was the primary end point per the IMWG criteria. The secondary end points included CR or better, DOR, PFS, OS, MRD, and safety. Patients received target doses of 150 × 10^6^, 300 × 10^6^, or 450 × 10^6^ CAR-positive T cells.

A total of 140 patients enrolled in the study and underwent leukapheresis. One hundred twenty-eight patients received ide-cel infusions. Of the treated patients, the median prior anti-myeloma regimens were six, and 94% had previously received auto-SCT; 35% had high-risk cytogenetics (del(17p), t(4;14), or t(14;16); 88% had received bridging therapy with a median duration of 15 days; 4% had a response to bridging therapy.

At a median follow-up of 13.3 months (0.2–21.2 months), 73% of treated patients (95% CI 66–81, p < 0.001) had a response, 52% had a very good partial response or better, and 33% of patients had a CR or stringent CR. Those who had a CR or stringent CR of 79% were MRD-negative (sensitivity level of 10^−5^). Overall response was seen in ≥50% of patients treated. This was consistently seen in most subgroups, including those with more aggressive disease features. The median time to first response was 1 month (0.5–8.8), and the median time to a CR or better was 2.8 months (1.0–11.8 months). The estimated median DOR overall by the Kaplan–Meier was 10.7 months (95% CI 9.0–11.3). The estimated PFS and OS by the Kaplan–Meier were 8.8 months overall (95% CI 5.6–11.6) and 19.4 months (95% CI 18.2–NE), respectively. The OS at 12 months was 78%.

Of treated patients, 99% had grade 3 or 4 AEs, with most grade 3 or 4 AEs being cytopenias; neutropenia was seen in 89%, anemia in 60%, and thrombocytopenia in 52%. Infections were observed in 69%, and 22% were grade 3 or 4 in nature.

CRS was seen in 84% of patients, and the majority was grade 1 or 2. Five patients had grade 3 CRS, one had grade 4, and one had grade 5. The median onset of CRS was 1 day, and the median duration was 5 days.

Neurotoxicity was seen in 18% of patients, and 3% had grade 3 events. No grade 4 or 5 neurotoxic events were seen. The median time to an observed neurotoxic effect was 2 days, with a median duration of 3 days (40).

KarMMa-3 was a phase 3, international, open-label, randomized study that evaluated ide-cel to standard regimens in patients with R/R MM. Patients had to have received two to four prior therapies that included daratumumab, an immunomodulatory agent, and a proteasome inhibitor for at least two consecutive cycles. Patients were randomized in a 2:1 fashion to receive either ide-cel or one of five standard regimens. Patients who were randomized to the SOC arm received one of the following SOC regimens per investigator’s discretion: daratumumab, pomalidomide, and dexamethasone; daratumumab, bortezomib, and dexamethasone; ixazomib, lenalidomide, and dexamethasone; carfilzomib and dexamethasone; or elotuzumab, pomalidomide, and dexamethasone.

The primary end point was PFS. The secondary end points included response, which was defined as PR or better, OS, time to response, DOR, detection of MRD, cellular kinetic and pharmacokinetic profiles, and safety.

Two hundred fifty-four patients were randomized to the ide-cel arm and 132 to the SOC arm. Two hundred fifty patients who were randomized to the ide-cel group ultimately were treated with ide-cel, and 126 of the SOC group received their treatment.

At a median follow-up of 18.6 months (0.4–35.4), PFS was significantly longer with ide-cel in comparison to the SOC group (p < 0.001), with a median of 13.3 months (95% CI 11.8–16.1) versus 4.4 months (95% CI 3.4–5.9), respectively. At 12 months, PFS was 55% in the ide-cel group and 30% in the SOC group. This PFS benefit was seen consistently across all subgroups. The ide-cel group had a significantly higher response to treatment in comparison to SOC, with 71% (95% CI 66–77) in the ide-cel group having a PR or better and 42% (95% CI 33–50) in the SOC group (95% CI 33–50, p < 0.001). The percentage of patients in the ide-cel group with CR or stringent CR was higher than with SOC, at 39% versus 5%, respectively. The median time to response was 2.9 months (0.5–13.0) in the ide-cel group versus 2.1 months (0.9–9.4) in the SOC group. The median duration was higher in the ide-cel group at 14.8 months (95% CI 12.0–18.6) versus that in the SOC group at 9.7 months (95% CI 5.4–16.3). Within 3 months prior to the occurrence of at least a CR, MRD negativity was seen in 20% of patients in the ide-cel group and 1% in the SOC group. The OS data were immature at the data cutoff.

AEs were seen in 99% and 98% in the ide-cel group and the SOC group, respectively; 93% of the ide-cel group had grade 3 or 4 AEs, and 75% of the SOC group had grade 3 or 4 AEs. Neutropenia was seen in 78% of patients in the ide-cel group and 44% in the SOC group. Infections were seen in 58% of the ide-cel group and 54% of the SOC group.

Of the ide-cel group, 88% had CRS. Most were grade 1 or 2 in nature (83%); 4% had grade 3 or 4 CRS, and 1% had grade 5 CRS. The median onset of CRS was 1 day, and the median duration was 3.5 days.

Neurotoxic events were seen in 15% in the ide-cel group. Most were grade 1 or 2 in nature (12%); 3% had a neurotoxic event that was grade 3 or higher. The median time to onset of a neurotoxic event was 3 days (41).

At extended follow-up with a median of 30.9 months, the PFS benefit of ide-cel in comparison to the SOC continued, with median PFS of 13.8 months (95% CI 11.8–16.1 months) and 4.4 months (95% CI 3.4–5.8), respectively. At 18 months, the PFS rate was 41% in the ide-cel group and 19% in the SOC group. There was a benefit of PFS seen in the ide-cel group in comparison to SOC across those with all prior lines of treatment; however, there was a greater benefit seen in those with earlier lines. Ide-cel also continued to show an improved ORR in comparison to SOC at 71% versus 42%, respectively. Similar safety data were seen in an extended follow-up. No new safety signals were observed (42).

#### 4.1.1 Real-world data

Real-world data evaluating ide-cel in R/R MM were evaluated using retrospective data across 11 US medical centers, with 159 patients. The median PFS at median follow-up of 6.1 months was 8.5 months (95% CI 6.5–NR), and the median OS was 12.5 months (95% CI 11.3–NR). CRR or better was 42%, and the best ORR was 84%. CRS of any grade was 82%, and grade 3 or higher was 3%. Any-grade neurotoxicity was seen in 18%, and grade 3 or higher was 6%. These safety and efficacy results were similar to those of KarMMa, and it did not show new safety signals (43).

### 4.2 Cilta-cel

CARTITUDE-1 was a phase 1/2, single-arm, open-label study evaluating the safety and clinical activity of ciltacabtagene autoleucel (cilta-cel) in adult patients with R/R MM. Cilta-cel is a CAR-T that expresses two BCMA-targeting single-domain antibodies with a 4-1BB costimulatory domain and CD3-ζ signaling domain. Patients were eligible if they received three or more lines of prior therapy or were double refractory to a proteasome inhibitor and an immunomodulatory drug, and if they were exposed to a proteasome inhibitor, immunomodulatory drug, and an anti-CD38 antibody. For phase 1 of the trial, the primary end point was incidence and severity of AEs, and for phase 2, it was ORR, which was defined by having a PR or better according to the IMWG criteria. The secondary end points included the rate of stringent CR, CR, and very good partial response (VGPR), DOR, MRD negativity, PFS, and pharmacokinetic and pharmacodynamic markers. One hundred thirteen patients were enrolled and had apheresis. Of those enrolled, 14% did not receive cilta-cel due to disease progression, death, or withdrawal from the study. Twenty-nine patients were in phase 1b, and 68 patients in phase 2 of the study (97 patients total) received cilta-cel at the dose of 0.75 × 10^6^ CAR-T cells per kg. High-risk cytogenetics was seen in 47%. The median lines of prior therapy were six. Bridging therapy was received by 75%. The median time from receiving cilta-cel to release of the product was 29 days.

The median time to first response from treatment was 1 month, and the median time to best response was 2.6 months. ORR at median follow-up of 12.4 months was 97% (95% CI 91.2–99.4); 67% of patients had stringent CR. The median DOR was NR (95% CI 15.9–NE) as well as median PFS (95% CI 16.8–NE). The 12-month PFS and OS were 77% (95% CI 66–84.3) and 89% (95% CI 80.2–93.5), respectively. These responses were similar across the subgroups of patients. Of the patients whose MRD status was able to be evaluated, 93% had MRD negativity.

Any-grade AE was seen in 100% of the patients, and 94% were grade 3 or 4 in nature. Of the AEs, hematologic AEs were the most common; 100% of patients had any-grade hematologic AE. This included neutropenia (95%), anemia (68%), leukopenia (61%), thrombocytopenia (60%), and lymphopenia (50%); 58% of patients had an infection, and 20% were grade 3–4 in nature.

CRS was seen in 95% of patients. Grade 1 CRS was seen in 51%, grade 2 in 39%, grade 3 in 3%, grade 4 in 1%, and grade 5 in 1%. The median time from cilta-cel infusion to onset of CRS was 7 days, and the median duration was 4 days. ICANS and other neurotoxicities were seen in 21% of patients, and ICANS alone occurred in 17% of patients; 10% had grade 1 ICANS, 4% had grade 2, 1% had grade 3, and 1% had grade 4. The median time to onset of ICANS was 8 days, and the median duration was 4 days. Neurotoxicities outside of ICANS included movement and neurocognitive treatment-emergent AEs. Of those who had another neurotoxic event, 50% had resolution of their symptoms. The median time to recovery was 74.5 days (44).

Long-term follow-up of CARTITUDE-1 was evaluated. The median follow-up was 61.3 months. The median OS was 60.7 months (95% CI 41.9–NE); 33% of the patients were alive and progression-free for 5 or more years; of these patients, 96.9% had stringent CR as their best response. This was the first instance in which there were data suggesting that cilta-cel is potentially curative in patients with R/R MM. No new safety signals were seen, with no new cases of parkinsonism or cranial nerve palsies (45).

CARTITUDE-4, a phase 3, open-label, randomized trial, evaluated cilta-cel in R/R MM for patients with lenalidomide resistance who received one to three prior lines of therapy. This included a proteasome inhibitor and an immunomodulatory drug. Cilta-cel was compared to SOC. The SOC group received pomalidomide, bortezomib, and dexamethasone (PVd) or daratumumab, pomalidomide, and dexamethasone (DPd) by the physician’s choice. The cilta-cel group received bridging therapy either with PVd or DPd. The primary outcome was PFS, and secondary outcomes included OS, MRD status, and overall response. Those with high-risk cytogenetics comprised 59.4% in the cilta-cel group and 62.9% in the SOC group. The median turnaround time for cita-cel was 44 days. In the intention-to-treat population, the PFS at 12 months was 75.9% (95% CI 69.4–81.1) for the cilta-cel group and 48.6% (95% CI 41.5-55.3) for the SOC group. The median duration for the cilta-cel group was NR, and for the SOC group, it was 11.8 months (95% CI 9.7–13.8); 73.1% of the cilta-cel group had a CR or better in comparison to 21.8% in the SOC group (p < 0.001). The overall response for cilta-cel was 84.6% and 67.3% with SOC (p < 0.001). Cilta-cel had a 60.6% MRD negativity in comparison to the SOC with 15.6% (p < 0.001). Cilta-cel was found to significantly decrease the risk of disease progression or death in comparison to SOC (p < 0.001).

Of the cilta-cel group, 96.6% had grade 3 or 4 AEs, and the SOC group had 94.2%. The most common grade 3 or 4 AEs seen in both the cilta-cel and SOC groups were hematologic. Infections were seen in 62% of the cilta-cel group and 71.2% of the SOC group, with 26.9% and 24.5% of grade 3 and 4, respectively. Of the cilta-cel group, 76.1% had any-grade CRS, with 75% having grade 1 or 2 and 1.1% having grade 3. The median time to onset of CRS was 8 days, and the duration was 3 days.

Any-grade neurotoxic event was seen in 20.5% of cilta-cel patients, grade 1 or 2 in 17.6%, and grade 3 or higher in 2.8%. Of the neurotoxic AEs, eight were ICANS, and they were all grade 1 or 2. The median onset of ICANS was 10 days, and the duration was 2 days. Cranial nerve palsies were seen in 9.1%, with the most commonly affected being cranial nerve VII. The median onset of cranial nerve palsy was 21 days, and 14 patients had recovered by the end of the data cutoff (46).

#### 4.2.1 Real-world data

Real-world data regarding cilta-cel in R/R were evaluated retrospectively across 16 US medical centers, with 236 patients. The median PFS was NR at median follow-up of 13 months, and the 12-month PFS estimate was 68% (95% CI 62–74). ORR was 89%, and CRR was 70%. CRS was seen in 75%, with 5% having grade 3 or higher, and ICANS was seen in 14%, with 4% having grade 3 or higher. Thus, similar results were seen in CARTITUDE (47) (**Table 3**).

**Table 3:** Overview of FDA Approved CAR-Ts in Hematologic Malignancies

Construct	Clinical trial	Targets	Diseases	Trial phase	Response rate (ORR/CRR)	Toxicities
Axi-cel	ZUMA-5	CD19	FL, MZL	Phase 2	92%/74%	CRS: any grade 84%, grade 3 or higher 7%
						ICANS: any grade 59%, grade 3 or higher 19%
	ZUMA-1	CD19	LBCL	Phase 1/2	82%/54%	CRS: any grade 93%, grade 3 or higher 13%
						ICANS: any grade 64%, grade 3 or higher 28%
	ZUMA-7	CD19	LBCL	Phase 3	83%/65%	CRS: any grade 92%, grade 3 or higher 6%
						ICANS: any grade 60%, grade 3 or higher 21%
Tisa-cel	ELARA	CD19	FL	Phase 2	86%/69%	CRS: any grade 49%, grade 3 or higher 0%
						ICANs: any grade 4%, grade 3 or higher 1%
	JULIET	CD19	LBCL	Phase 2	34%/40%	CRS: any grade 58%, grade 3 or higher 22%
						ICANS: any grade 21% grade 3 or higher 12%
	ELIANA	CD19	B-ALL	Phase 2	82%/60%	CRS: any grade 77%, grade 3 or higher 46%
						ICANS: any grade 40%, grade 3 or higher 13%
Liso-cel	TRANSCEND FL	CD19	FL	Phase 2	3L+ 97%/94%; 2L 96%/100%	2L CRS: any grade 52%, grade 3 or higher 0% ICANS: any grade 17%, grade 3 or higher 4% 3L+ CRS: 58%, grade 3 or higher ~1% ICANS: any grade 15%, grade 3 or higher 2%
	TRANSCEND CLL	CD19	CLL	Phase 1/2	44%/20%	CRS: any grade 85%, grade 3 or higher 9%
						ICANS: any grade 45%, grade 3 or higher 18%
	TRANSCEND NHL 001	CD19	LBCL	Phase 1	73%/53%	CRS: any grade 42%, grade 3 or higher 2%
						ICANS: any grade 30%, grade 3 or higher 10%
	PILOT	CD19	LBCL	Phase 2	80%/54%	CRS: any grade 38%, grade 3 or higher 2%
						ICANS: any grade 31%, grade 3 or higher 5%
	TRANSFORM	CD19	LBCL	Phase 3	86%/66%	CRS: any grade 49%, grade 3 or higher 1%
						ICANS: any grade 12%, grade 3 or higher 4%
	TRANSCEND MCL	CD19	MCL	Phase 1	87%/74%	CRS: any grade 61%, grade 3 or higher 1%
						ICANS: any grade 31%, grade 3 or higher 9%
Obe-cel	FELIX	CD19	B-ALL	Phase 1b/2	77%/55%	CRS: any grade 68%, grade 3 or higher 2%
						ICANS: any grade 22%, grade 3 or higher 7%
Brexu-cel	ZUMA-2	CD19	MCL	Phase 2	85%/59%	CRS: any grade 91%, grade 3 or higher 15%
						ICANS: any grade 63%, grade 3 or higher 31%
	ZUMA-3	CD19	B-ALL	Phase 2	71%/56%	CRS: any grade 89%, grade 3 or higher 24%
						ICANS: any grade 60%, grade 3 or higher 26%
Ide-cel	KarMMa	BCMA	MM	Phase 2	73%/33%	CRS: any grade 84%, grade 3 or higher 5%
						ICANS: any grade 18%, grade 3 or higher 3%
Cilta-cel	CARTITUDE-1	BCMA	MM	Phase 1b/2	97%/69%	CRS: any grade 95%, grade 3 or higher 5%
						ICANS: any grade 17%, grade 3 or higher 2%

ORR, overall response rate; CRR, complete remission rate; Axi-cel, axicabtagene ciloleucel; Tisa-cel, tisagenlecleucel; Liso-cel, lisocabtagene maraleucel; Obe-cel, obecabtagene autoleucel; Brexu-cel, brexucabtagene autoleucel; Ide-cel, idecabtagene vicleucel; Cilta-cel, ciltacabtagene autoleucel; FL, follicular lymphoma; MZL, marginal zone lymphoma; CRS, cytokine release syndrome; LBCL, large B-cell lymphoma; B-ALL, acute lymphoblastic B-cell leukemia; MM, multiple myeloma.

## 5 Resistance mechanisms in CAR-T

Both primary and secondary resistance can be seen. Primary resistance is when patients do not respond to treatment, while secondary resistance is when patients have a relapse after an initial response. One mechanism of resistance seen in CAR-T includes T-cell exhaustion. In the setting of auto-CAR-T, T cells come from the patients. Many of these patients have experienced multiple lines of therapy, which can lead to T-cell exhaustion. It can also occur after prolonged antigen exposure. The fitness of the T cells at the time of infusion impacts potential response to therapy. It can also affect their expansion capacity and decrease their cytotoxic ability. Antigen escape is another potential method of resistance in which loss of target antigen expression can lead to immune escape from CAR-T therapy. The immunosuppressive nature of the tumor microenvironment can also influence CAR-T efficacy and lead to resistance. There have been strides to help overcome these resistance mechanisms with novel approaches (48).

## 6 Novel CAR-Ts

### 6.1 Armored CAR-T

A method to try to improve CAR-T is using armored CAR-T cells. These CAR-T cells are engineered to overcome the immunosuppressive tumor microenvironment that can be seen in malignancies and lead to resistance. Armored CAR-T cells can secrete an antibody-like protein to help target a tumor antigen, a proinflammatory cytokine that helps increase anti-tumor activity, or express a cytokine receptor to help change the cytokine environments seen in the tumor microenvironment. These cytokines include interleukin (IL) 12, IL-18, IL-15, IL-17, IL-4, and IL-7. An example of an armored CAR-T is huCART19-IL18, which is an autologous CD19-targeted CAR-T that releases IL-18. It is also manufactured quickly (3-day process) to attempt to keep its stem cell-like characteristics and potentially decrease T-cell exhaustion (49). In a phase 1 trial, huCART19-IL18 was administered to 21 patients who had CD19-positive B-cell lymphomas. The median lines of prior therapies were seven, with a range of 4–14. CRS was seen in 62% of patients, with 47% having grade 1 or 2. ICANS was seen in 14%, with all grade 1 or 2 in nature; 14% had grade 3 infections. Three months after receiving huCART19-IL18, 81% (90% CI 62–93) had either CR or PR, and 52% had CR (90% CI 33–71). At median follow-up of 17.5 months, the median DOR was 9.6 months (90% CI 5.5–NR) (50).

### 6.2 *In vivo* CAR-T

A method of CAR-T production that is currently being explored is *in vivo* CAR-T. The current typical manufacturing of CAR-T is *ex vivo*, in which T cells are extracted and isolated, and then modified and expanded outside of the patient’s body. However, with *in vivo* CAR-T, the CAR constructs are made within the patient’s body. This occurs with either viral vectors or nanocarriers carrying a CAR gene editing construct, which then targets T cells and causes expression of the CAR construct on the T-cell surface. The benefits of *in vivo* CAR-T are potential cost reduction, faster turnaround times, more convenience, and the elimination of the need for lymphodepleting chemotherapy. There are limitations with this CAR-T production method, which include potential off-target effects, issues with stability and persistence of the viral vectors/nanocarriers, and appropriate expansion of the CAR constructs (51).

### 6.3 Allo-CART

Allogenic CARTs (allo-CARTs) differ from auto-CARTs, as they are produced from a healthy donor’s T cells that can allow an off-the-shelf option and help avoid T-cell exhaustion that can be seen in auto-CARTs. However, a potential disadvantage of this therapy is graft-versus-host disease (GVHD), which occurs when the allo-CART cells recognize a patient’s healthy normal tissues by a T-cell receptor (TCR) and attack them. The primary T cells that are collected by leukapheresis are divided into two groups: αβ T cells and γδ T cells. αβ T cells comprise approximately 95% of the circulating T cells, and they can recognize antigens by their TCRs through Human Leukocyte Antigens (HLAs). Thus, these T cells can cause GVHD. To avoid GVHD, the T cells obtained from the donor are knocked out *ex vivo* for a reactive antigen such as TCR or HLA. This can be performed using gene editing tools such as CRISPR-Cas9 or TALEN. Alternatively, γδ T cells have γδ T-cell receptors that identify target antigens without the use of HLA presentation. Therefore, it can be a potential source of allo-CART without the potential risk of GVHD. However, it is challenging to generate allo-CARTs using γδ T cells, as they typically comprise approximately 5% of T cells in the peripheral blood. Currently, there are numerous clinical trials evaluating allo-CARTs in hematologic malignancies that appear to be promising and may be the standard of care in the future (52).

### 6.4 Dual-target CAR-T

There has been movement toward dual- or triple-targeted CAR-T to recognize two or three antigens rather than the standard single antigen. The goal would be to overcome potential antigen escape that can be seen with single-target CAR-Ts and minimize potential on-target/off-tumor toxicities. Novel dual-target CAR-Ts include CD19/CD20 and CD19/CD22 in NHL and B-ALL, and CD19/BCMA and CD38/BCMA in MM. There were pre-clinical data showing the superior efficacy of dual-target CAR-T cells in comparison to single-target CAR-T cells. This has not clearly translated into clinical practice. A phase 1 clinical trial comparing BCMA/CD19 CAR-T versus BCMA-only CAR-T in MM did not show improved efficacy. However, a trial that evaluated CD19/22 CAR-T versus CD19-only CAR-T in B-ALL showed an improved CRR. There have been some reports of further antigen loss seen with dual-target CAR-Ts. Further investigation of dual-target CAR-Ts is needed, and there are active clinical trials exploring this novel CAR-T approach (53).

## 7 Discussion

CAR-T therapy has revolutionized the hematologic malignancy treatment space. There are now seven food and drug administration (FDA)-approved CAR-Ts for NHL, B-ALL, and MM from the results of the clinical trials outlined above, which have also translated into real-world data. The addition of CAR-T in R/R LBCL led to an improvement in OS in comparison to SOC and caused a paradigm shift in its treatment. In MM, long-term data regarding cilta-cel showed that one-third of patients remain disease-free 5 years from treatment, which suggests a possible cure. This highlights the significant impact CAR-T has had in this field. There have also been strides to improve these SOC CAR-Ts to attempt to overcome resistance mechanisms. This includes dual-target CAR-Ts and allo-CARTs to help combat antigen escape or T-cell exhaustion, respectively. These novel CARs are currently being studied, and while some have shown promising results, some have been found to have their own shortcomings and difficulties in translating clinically. There continue to be advancements in CAR-T therapies with promising developments that may bring about a new era in the management of hematologic malignancies.
